# A Wideband Cryogenic Readout Amplifier with Temperature-Insensitive Gain for SNSPD

**DOI:** 10.3390/s22031225

**Published:** 2022-02-06

**Authors:** Xiaokang Niu, Lianming Li, Xu Wu, Dongming Wang

**Affiliations:** 1 National Mobile Communications Research Laboratory, Southeast University, Nanjing 210000, China; niuxiaokang@seu.edu.cn (X.N.); lianming.li@seu.edu.cn (L.L.); xu.wu@seu.edu.cn (X.W.); 2 Purple Mountain Laboratory, Nanjing 210000, China

**Keywords:** cryogenic amplifier, superconducting nanowire single photon detectors, SNSPD, wideband, readout, low power

## Abstract

This paper presents a temperature-insensitive wideband cryogenic amplifier for superconducting nanowire single-photon detectors (SNSPD). With a proposed folded diode-connected transistor load to realize a good device-tracking feature, the theoretical derivations the simulations and test results prove that the amplifier-gain cell has a stable gain performance over a wide temperature range, solving the issues of a lack of the accurate cryogenic device models. The amplifier achieves a gain of 26 dB from 100 kHz to 1 GHz at 4.2 K, consuming only 1.8 mW from a 1.8 V supply. With a 0.13-μm SiGe BiCMOS process, the chip area is 0.5 mm².

## 1. Introduction

**Figure 1:**
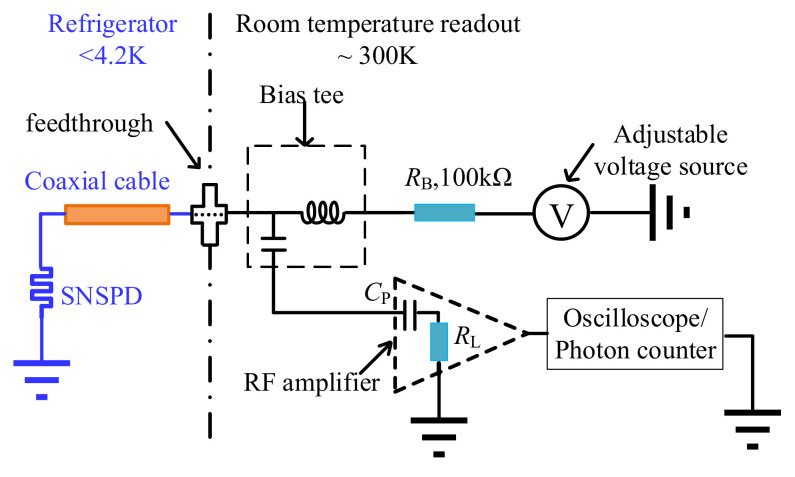
The traditional SNSPD readout scheme.

As key enabling components, SNSPDs [1,2] play an important role in the applications of high-speed quantum key distribution (QKD) [3,4], light detection and ranging (LIDAR) [5], high-sensitivity bio-medical imaging [6,7,8,9], remote sensing and detection [10]. To detect weak electrical SNSPD output signals [11], a readout amplifier with high gain and low noise performance is needed. As the traditional readout amplifiers have relatively large sizes and high-power consumption, they are difficult to assemble with SNSPDs. For illustration, as shown in Figure 1, the readout amplifier works at room temperature, and it is connected to the SNSPD in the cryogenic environment with a long coaxial cable. With tests, on one hand it is found that the long coaxial cable limits the size of the SNSPD readout system, making it difficult to realize compact multi-element SNSPD arrays. On the other hand, this large readout system would deteriorate the signal quality and introduce large signal jitters.

**Figure 2:**
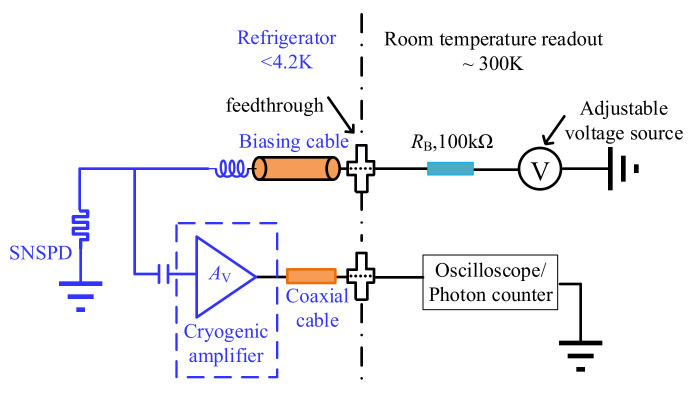
The cryogenic SNSPD readout scheme.

Considering the above issues, it is preferable to place the readout amplifier in a cryogenic environment, resulting in a compact cryogenic SNSPD readout system, as shown in Figure 2. In this way, the interconnecting line between the SNSPD and the cryogenic readout amplifier can be reduced substantially, reducing the insertion loss and signal interference. From the implementation perspective, due to the limited cooling capacity of the cryogenic refrigerator, the power consumption of the cryogenic amplifier should be low enough. The traditional radio frequency (RF) amplifiers with topologies such as Doherty and balanced structures exhibit remarkable performance in terms of power efficiency, gain, bandwidth and linearity [12,13], but they are not specifically designed for cryogenic applications. Different from these amplifiers, the cryogenic amplifier prefers higher input impedance, instead of conventional 50 ohm impedance, to obtain higher SNSPD signal voltage amplitude for a better signal-to-noise ratio [14,15]. Recently, several cryogenic readout amplifiers have been realized using III-V and silicon processes. A HEMT 0.5~13 GHz low noise amplifier with a gain of 38~44 dB is presented in [16]. However, this amplifier dissipates a high power consumption of 15 mW, making it difficult to be placed in the cryogenic environment. In [17,18], the power consumption of the realized amplifiers is lower than 1 mW, but their working frequencies are 4~8 GHz, which is not feasible for SNSPD readout applications. Note that compared with the SiGe process, the III-V process has the disadvantage of a low integration level and high cost. In [19], based on the Cherry-Hooper amplifier topology, a wideband cryogenic amplifier is implemented with the SiGe process, demonstrating a bandwidth of 3.4 GHz, with a power consumption of 4.3 mW. However, due to the high sensitivity of bipolar transistor transconductance to the operating temperature and the lack of accurate cryogenic device models, the cryogenic amplifier gain performance cannot be predicated easily in the design and simulation, resulting in the need for additional biasing tuning to assure the proper working of the amplifier.

To address the above issues, this paper proposes a robust temperature-insensitive gain-cell structure with a folded diode-connected transistor load. With calculations and simulations, it is proved that the gain cell has a stable gain across a wide temperature range due to its good device-tracking feature. With a 0.13 μm SiGe BiCMOS process, the test results show that the amplifier achieved a 26 dB gain from 100 kHz to 1 GHz at 4.2 K, while consuming only 1.8 mW without complex biasing tuning. 

The paper is organized as follows. Section 2 presents the amplifier requirements and topology. Section 3 explains the design detail of the temperature-insensitive gain cell. The circuit implementation, simulation and measurement results are described in Section 4. Finally, a conclusion is provided in Section 5.

## 2. The Amplifier Requirements and Topology

**Figure 3:**
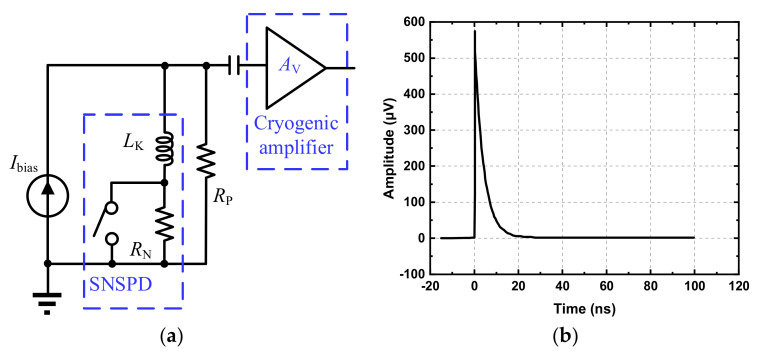
The connection of the SNSPD and readout amplifier, and output signal: (**a**) The SNSPD model and its connection with the amplifier; (**b**) Typical SNSPD output pulse signal.

For the proper design of the readout amplifier, it is important to evaluate the SNSPD readout requirements. For illustration, Figure 3a shows that the SNSPD is AC-coupled to the amplifier using a capacitor. With a current bias, typically the working behavior of the SNSPD can be described with an equivalent electrical lumped model. As shown in Figure 3a, this lumped model consists of a kinetic inductor *L*_K_, a resistor *R*_N_ and a switch [20]. To establish a proper current loop and to quench the device, a parallel resistor *R*_P_ with 50 Ohm is added [21]. Note that as the kinetic inductor *L*_K_ is much larger than its Faraday inductance and the parasitic capacitance is relatively small, the parasitic capacitance is ignored without losing the model accuracy, and the time constant of the transient pulse is mainly determined by the kinetic inductor *L*_K_ and corresponding resistance. When the SNSPD is in the superconducting state, the switch is closed. In contrast, when an incident photon is absorbed by the SNSPD, the switch is opened, pushing the current into the parallel resistor *R*_P_. Accordingly, a pulse voltage signal, typically with several hundred microvolts amplitude, is generated [22], as shown in Figure 3b. Normally, its rising time is around 200 ps, while its falling time ranges from 10 to 60 ns. To amplify such a pulse signal with high fidelity, the amplifier bandwidth should be wide enough. Otherwise the signal edge performance will be degraded, introducing several non-idealities and even leading to a wrong detection.

**Figure 4:**
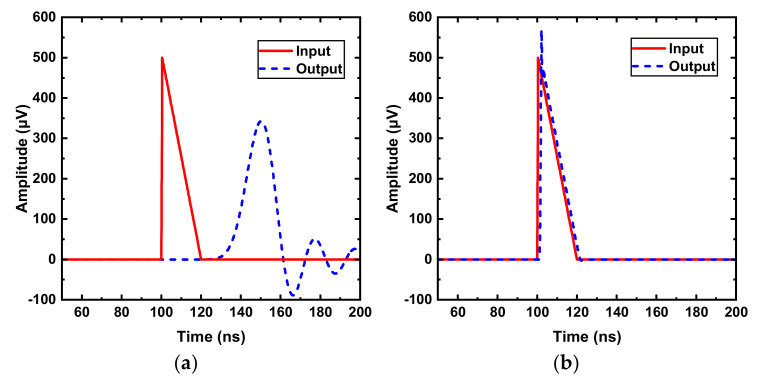
Transient response of the signal with different bandwidths: (**a**) Bandwidth = 100 MHz; (**b**) Bandwidth = 1 GHz.

To evaluate the bandwidth requirement of the amplifier, with an ideal unity gain amplifier, the following simulations were undertaken with different bandwidth conditions. As shown in Figure 4a,b, the solid lines represent the SNSPD input pulse signal with a 200 ps rising time and 20 ns falling time, while the dashed lines are the output signals after passing through the unity gain amplifier. As indicated, when the amplifier bandwidth is 100 MHz, the output signal has a significant delay of about 20 ns and experiences ringing issues. As shown in Figure 4b, when the bandwidth is increased to 1 GHz, the output agrees well with the input signal, with a delay of only 1.5 ns, which is smaller than 1/10 pulse width, meeting the system requirement. Note that a larger bandwidth is better for the signal fidelity, but it would introduce more noise and the amplifier dissipates more power. Considering these design trade-offs, in this work, the high cut-off frequency of the amplifier is set to around 1 GHz. Moreover, considering the issues of metastability, noise, etc., the minimum input signal of a high speed comparator needs to be higher than 5 mV [23] for a good BER (bit error rate) performance. With a typical amplitude of a SNSPD output signal of about 600-μV, the gain of the readout amplifier should be larger than 20 dB [19,24,25], thereby assuring the output signal swing is large enough for the post ADC processing circuitry. 

**Figure 5:**
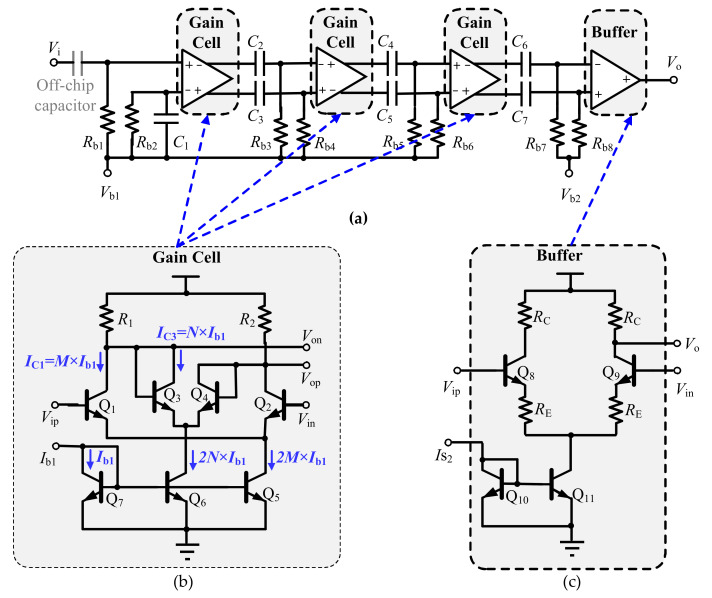
The proposed 4-stage cryogenic amplifier: (**a**) The amplifier topology; (**b**) Temperature-insensitive gain cell; (**c**) Output buffer stage.

To achieve sufficient gain performance, in this paper, a 4-stage cryogenic amplifier is proposed, as each stage has a limited gain. Figure 5a shows the topology of the proposed wideband bipolar cryogenic amplifier, which consists of three temperature-insensitive gain cells and an output buffer stage, shown in Figure 5b,c, respectively. Note that, the differential structures are used in these gain cells to improve the common-mode rejection ratio, making the amplifier robust to interference. 

As indicated in Figure 5a, its input gain cell is a kind of pseudo-differential configuration. By terminating the parallel resistor *R*_b2_ and capacitor *C*_1_ to ground, the single-ended to differential signal conversion is realized. To achieve better large signal performance and a better driving capability, an emitter degeneration structure is employed in the output buffer stage to drive the standard 50 Ohm impedance of the measurement equipment. Considering the lack of an accurate cryogenic device model, all the gain cells and the output buffer are AC-coupled to increase the DC biasing flexibility and to remove the DC offset from differential signals.

## 3. The Temperature-Insensitive Gain Cell Design

**Figure 6:**
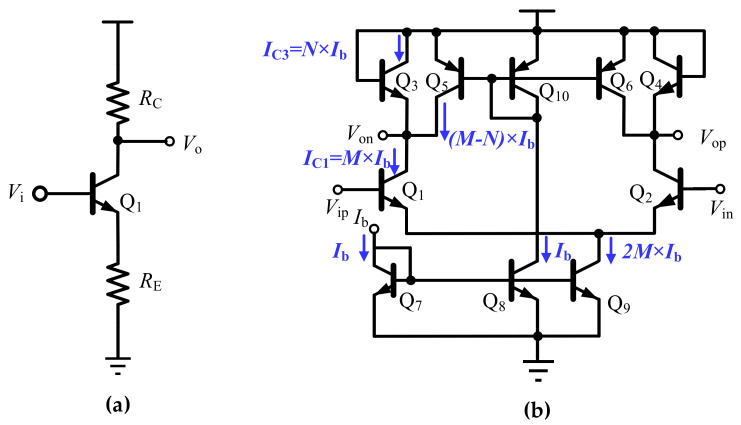
Schematic of (**a**) the typical common emitter gain cell with emitter degeneration; (**b**) the gain cell with the conventional diode-connected transistor load.

As mentioned before, bipolar transistors are sensitive to the temperature and its gain will change noticeable across a large temperature range. To solve this issue, a common-emitter gain cell topology with an emitter degeneration resistor can be used, as shown in Figure 6a. When the product of transistor Q_1_ transconductance and resistor *g*_m1_*R*_E_ is significantly larger than 1, the voltage gain of this cell is equal to the resistor ratio of *R*_C_/*R*_E_, which is insensitive to temperature variation. However, it should be noticed that, different to the thermal noise, the transistor shot noise does not scale with the temperature, and this gain cell suffers from large shot noise issues in the cryogenic environment. As a result, this emitter degeneration topology is not suitable for the input stage of the cryogenic amplifier, and it is employed as the output buffer with the merits of temperature-insensitive gain in this design, as shown in Figure 5c.

Figure 6b shows another gain-cell topology, in which the conventional diode-connected transistors, Q_3_ and Q_4_, are realized as the load. As to be shown shortly, its gain is defined by the ratio of transistor Q_1_ and Q_3_ transconductances, i.e., *g*_m1_ and *g*_m3_. For proper biasing, the current mirror with PNP transistor Q_5_, Q_6_ and Q_10_ is used to sink currents. 

**Figure 7:**
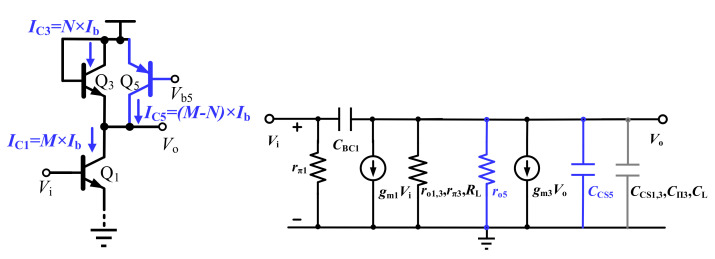
The simplified single-ended equivalent circuit and small signal model of the conventional gain cell.

To illustrate the working mechanism of the gain cell in Figure 6b, for cascading the amplifier design and analysis convenience, taking into account the Miller capacitor and loading effects, Figure 7 shows the simplified single-ended equivalent circuit and its small signal model. Supposing the biasing current through Q_7_ is *I*_b_, with a proper current mirror size ratio, the collector currents through transistors Q_1_ and Q_5_ are *M* × *I*_b_ and (*M − N*) × *I*_b_, respectively. As a result, the current through transistor Q_3_ is equal to *N* × *I*_b_.

The gain of the circuit is derived as follows:

(1) $$A_{v}=\frac{V_{o}}{V_{i}}=g_{m1}\left(\frac{1}{g_{m3}}\left\|r_{o5}\left\|r_{o1}\left\|r_{\pi 3}\right.\left\|r_{o3}\left\|R_{L}\right.\right.\right.\right.\right)$$

where *r*_o1,3,5_ and *r*_π1,3_ are the output and input resistance of the transistors, respectively, and *R*_L_ is the input resistance of the transistor from the following gain stage. By definition, transconductance of the bipolar transistor Q_3_ is given by

(2) $$g_{m3}=M\times I_{b}\frac{q}{kT}$$

Equation (2) reveals the transistor transconductance strongly depends on its biasing current and operating temperature *T*. When the biasing current increases or the operating temperature decreases, *g*_m3_ of the load transistor Q_3_ consequently increases. If the following condition is satisfied:

(3) $$\frac{1}{M\times I_{b}\frac{q}{kT}}<<r_{o5}\left\|r_{o1}\left\|r_{\pi 3}\right.\left\|r_{o3}\left\|R_{L}\right.\right.\right.$$

1/*g*_*m*3_ would dominate the load impedance, and the gain of the circuit can be rewritten as follows:

(4) $$A_{v}\approx g_{m1}\frac{1}{g_{m3}}=\frac{M\times I_{b}\frac{q}{kT}}{N\times I_{b}\frac{q}{kT}}=\frac{M}{N}$$

Accordingly, *A_v_* is equal to the current mirror ratio of *M*/*N*, and is not affected by the particular value of the biasing current *I*_b_ or temperature *T*. In other words, the gain performance of this gain cell becomes insensitive to the temperature. 

The bandwidth is also derived as follows:

(5) $${\mathrm{BW}}_{-3\mathrm{dB}}\approx \frac{1}{2\pi \frac{1}{g_{m3}}\left(C_{\mathrm{CS}1}+C_{\pi 3}+C_{\mathrm{CS}3}+C_{\mathrm{CS}5}+C_{L}+(1+\frac{1}{A_{v}})C_{\mathrm{BC}1}\right)}\approx \frac{q}{2\pi kT}\frac{N\times I_{b}}{\left(C_{\mathrm{CS}1}+C_{\pi 3}+C_{\mathrm{CS}3}+C_{\mathrm{CS}5}+C_{L}+C_{\mathrm{BC}1}\right)}$$

where *C*_CS1,3,5_ and *C*_π3_ are the output and input parasitic capacitance of the transistors, respectively. Additionally, *C*_BC1_ is the Miller capacitance of the transistors Q_1_, and *C*_L_ includes the capacitance from Miller effect and input capacitance of the following gain stage. However, due to the large parasitic capacitance *C*_CS5_ contributed by the PNP transistors Q_5_, the bandwidth of the gain cell is reduced, as to be shown shortly.

**Figure 8:**
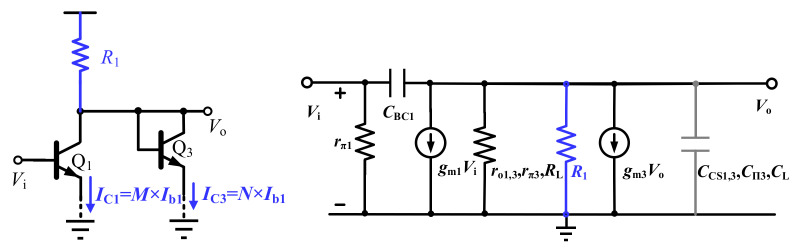
The simplified single-ended equivalent circuit and small signal model of the proposed gain cell with folded diode-connected load.

Considering the above-mentioned issues, the diode-connected transistors Q_3,4_ are folded in this design, leading to the proposed temperature-insensitive gain cell, as shown in Figure 5b. Different from the conventional diode-connected gain cell topology, correct DC paths are established by the resistor R_1,2_ and the tail current sources Q_5,6_. With a biasing current *I*_b__1_ and a proper current–mirror ratio, the currents through transistor Q_1_ and Q_3_ are *M* × *I*_b__1_ and *N* × *I*_b__1_, respectively. In this way, the PNP transistors in Figure 6b can be removed. For calculation convenience, Figure 8 shows the equivalent small signal model of the proposed gain cell.

Similar to the theoretical derivations above, the gain and the bandwidth of the proposed gain cell are given by

(6) $$A_{v}=g_{m1}\left(\frac{1}{g_{m3}}\left\|R_{1}\left\|r_{o1}\left\|r_{\pi 3}\right.\left\|r_{o3}\right.\right.\right.\left\|R_{L}\right.\right)\approx g_{m1}\frac{1}{g_{m3}}=\frac{M}{N}$$

(7) $${\mathrm{BW}}_{-3\mathrm{dB}}\approx \frac{q}{2\pi kT}\frac{N\times I_{b1}}{\left(C_{\mathrm{CS}1}+C_{\pi 3}+C_{\mathrm{CS}3}+C_{L}+C_{\mathrm{BC}1}\right)}$$

Note that the value of R_1,2_ is chosen to be significantly larger than 1/*g*_m3,4_, and it is realized by the ploy resistor with low temperature coefficient [26,27]. With simulations, it is proved that the amplifier bandwidth and gain change are slightly even with ±35% resistance variation. Clearly, due to the good device tracking feature of NPN transistors, the voltage gain *A*_v_ of the proposed gain cell is determined only by the current mirror ratio *M/N*, which is three in this design. Equation (6) is valid both at room and cryogenic temperature when the transistor Q_3_ transconductance dominants the load impedance, and the gain cell can achieve a constant gain at cryogenic temperature. It avoids the need of an accurate cryogenic device model during the design process.

**Figure 9:**
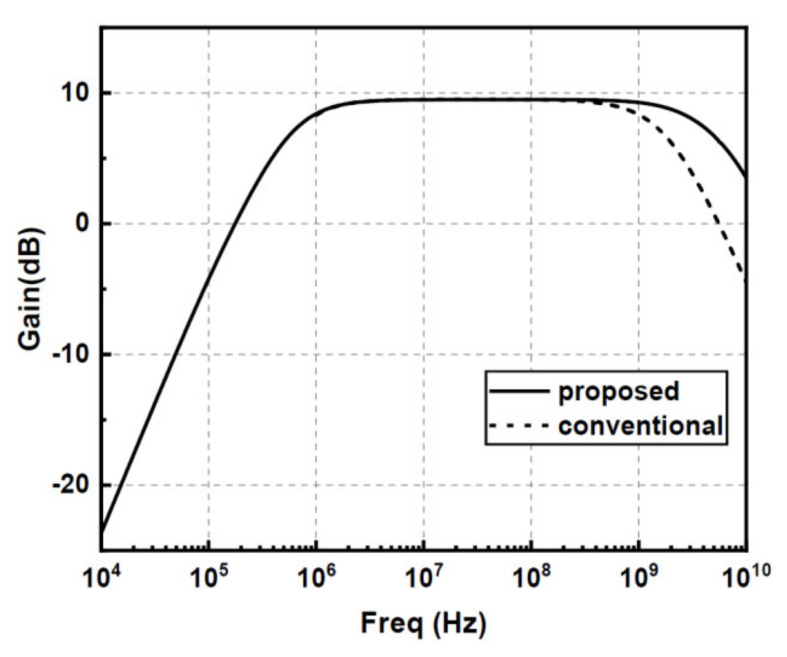
The simulated frequency response results of the proposed and the conventional gain cell.

Figure 9 compares the frequency responses of the conventional diode-connected gain cell and the proposed folded diode-connected gain cell at room temperature. Clearly, with M/N ratio of three, both gain cells achieve a gain of 9.5 dB, which agrees well with the theoretical calculation results. With the benefit of removing the large PNP transistor parasitic capacitance, the proposed folded diode-connected gain cell exhibits a higher bandwidth than the conventional cell with the same biasing current of 60-μA.

**Figure 10:**
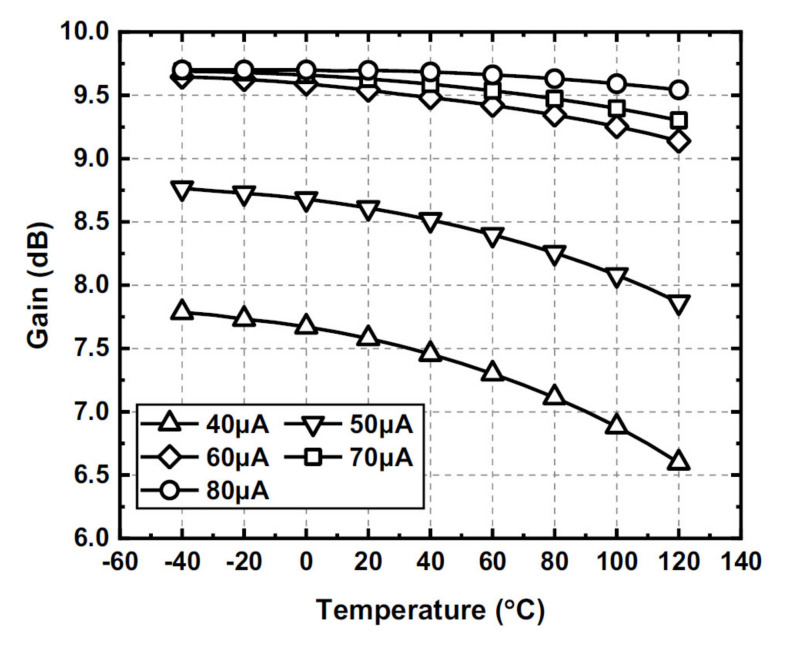
The simulated gain at 100 MHz of the proposed gain cell with different operating temperatures and biasing currents.

As the valid temperature range for the commercial model of the SiGe process is from −40 to 120 °C, to further illustrate the working mechanism of the proposed gain cell with different temperature and biasing current, in this design the amplifier gain performance at 100 MHz is simulated with the above temperature range with a biasing current *I*_b1_ ranging from 40 to 80 μA, as shown in Figure 10. 

As indicated in Figure 10, at a particular temperature, the gain of the amplifier gradually approaches the saturation gain value of about 9.5 dB as the biasing current *I*_b1_ increases. On the other hand, as the operating temperature decreases, the gain cell can reach a saturation value with a smaller biasing current *I*_b1_. The transistor transconductance increases substantially when the temperature drops to a cryogenic temperature, even with a smaller biasing current [26]. Therefore, the transistor Q_3_ transconductance still dominates the load impedance even at cryogenic temperature, allowing the amplifier to achieve a saturation gain value.

The results can be understood and justified as follows: by increasing the biasing current or reducing operating temperature *T*, the transistor transconductance increases and dominates the load impedance; therefore, the gain reaches the saturation gain value close to the ratio *M*/*N* of three, which is not affected by temperature variation. Therefore, the effectiveness of Equation (6) is proven, and the saturated gain performance of the gain becomes insensitive to temperature variation. With the above observations, it can be predicted that the biasing current required to achieve the saturated gain value can be reduced substantially at a cryogenic temperature.

Due to the lack of a cryogenic device model, for amplifier noise optimization, the noise simulation was undertaken at room temperature, showing that the equivalent integrated input referred noise amplitude within the passband from 1 MHz to 1 GHz is less than 70 μV. This input referred noise amplitude is significantly smaller than typical SNSPD input signal amplitude. As highlighted by [28], the transistor and the amplifier noise decreased substantially at cryogenic temperature, and it can be predicted that the cryogenic amplifier has good noise performance for SNSPD-readout applications. 

## 4. Implementation, Simulation and Measurement Results

**Figure 11:**
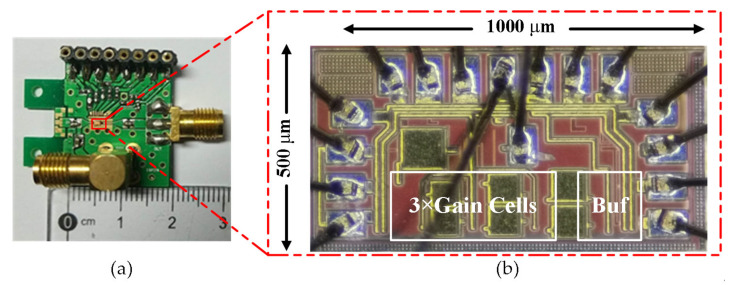
The photograph of PCB test board and the cryogenic amplifier chip: (**a**) The PCB board; (**b**) Chip microphotograph.

With the benefit of high integration and low cost, the proposed amplifier is fully integrated in a single chip and fabricated with a 0.13 μm SiGe BiCMOS process, which provides a high performance NPN transistor with a peak *f*_T_ of 210 GHz, poly resistors, metal-insulator-metal (MIM) capacitors and seven metal layers. Figure 11a shows the realized PCB test board, and the SMA connectors are used for input and output ports. Figure 11b shows the amplifier die microphotograph and the chip area is about 1 mm × 0.5 mm. The amplifier measurements were undertaken at room and cryogenic temperatures, respectively.

### 4.1. Room Temperature Performance

**Figure 12:**
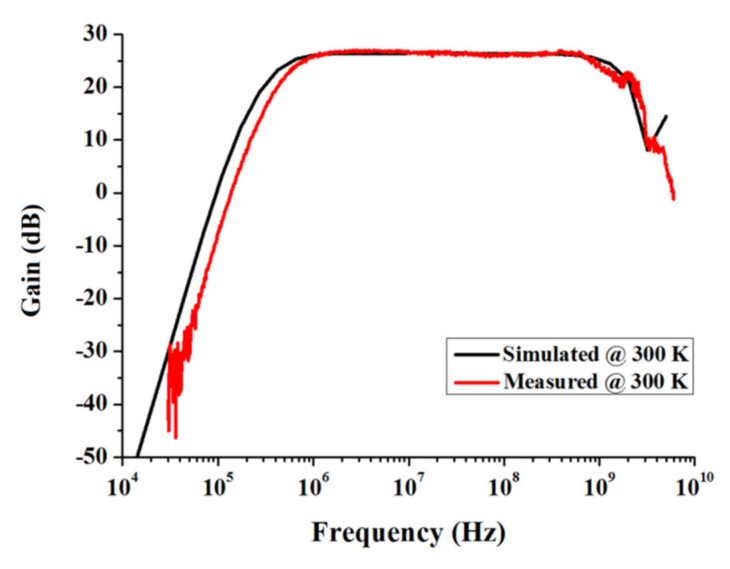
Simulated and measured gain of the amplifier at 300 K.

With the biasing voltage *V*_B1_ and *V*_B2_ of 1.5 V, the amplifier is biased with *I*_b1_ and *I*_b2_ of 60 and 150 µA, respectively, and consumes a current of 4.5 mA from a 1.8 V supply voltage. Measured with a network analyzer, Figure 12 shows the measured gain at 300 K room temperature. As indicated, the amplifier achieved a gain of 26 dB with bandwidth over 1 GHz. Moreover, the amplifier measurement results are compared with the simulation results under the same biasing condition, showing good agreement with each other. Note that due to the bonding wire parasitic inductance, some gain ripples were introduced at a high frequency. 

### 4.2. Cryogenic Performance

**Figure 13:**
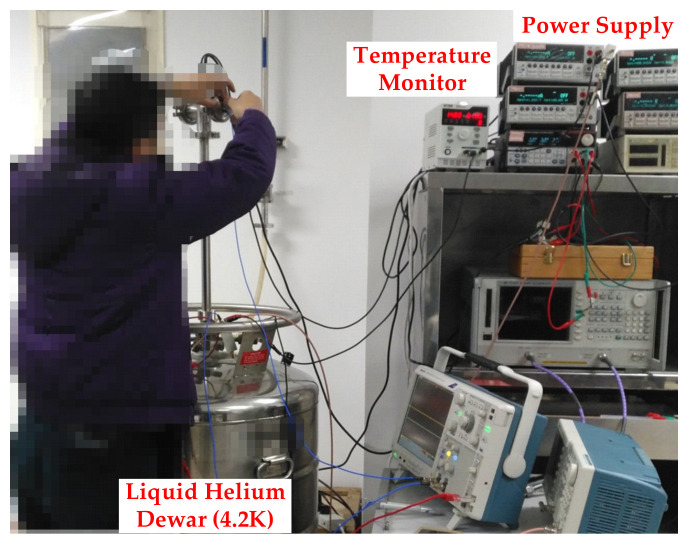
The measurement setup at cryogenic temperature.

Figure 13 shows the measurement setup at cryogenic temperature. With the help of the special Dewar containing the liquid helium, the amplifier was cooled down to the specific temperature of 4.2 K. Note that two chips are tested in this work and their results are quite close to each other. For clearer illustration, only one of the chip results are shown in the following parts.

**Figure 14:**
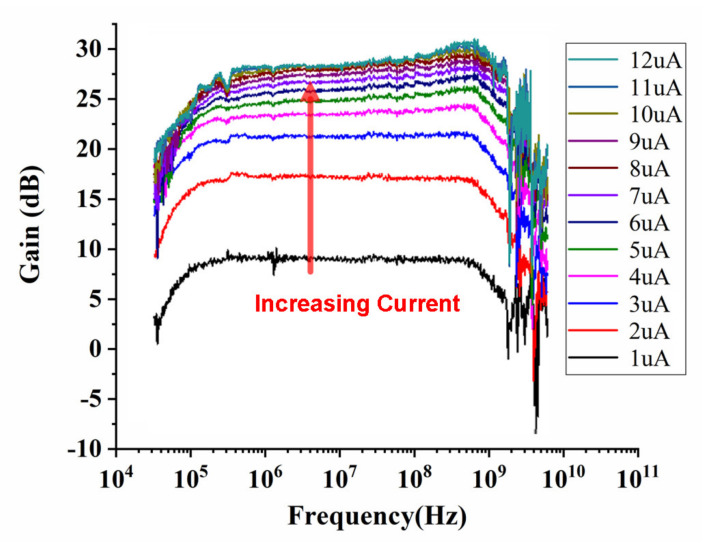
Measured gain with different biasing current *I*_b1_ ranging from 1 to 12 μA at 4.2 K.

With the same biasing voltage *V*_B1_ and *V*_B2_ of 1.5 V at 300 K, Figure 14 shows the measured gain with a biasing current *I*_b1_ ranging from 1 to 12 μA, while *I*_b2_ is set to 50 μA. As illustrated, the amplifier gain improved with increased biasing current *I*_b1_. When the biasing current *I*_b1_ was larger than 9 μA, the amplifier gain was saturated to 26 dB. Compared with Figure 12, it can be seen that the amplifier achieved the same saturated gain at 4.2 K and 300 K. It also indicates that, when the temperature decreased dramatically to 4.2 K, with a lower biasing current, the amplifier still achieved a predicable saturated gain even without an accurate cryogenic device model. With such advantages, the cryogenic amplifier consumes 1.8 mW with *I*_b1_ = 9 μA, which is only 22% of the amplifier power consumption at room temperature. These results agree well with the theoretical analysis, simulation analysis and prediction in Section 3, proving the effectiveness of the proposed temperature-insensitive gain cell.

**Figure 15:**
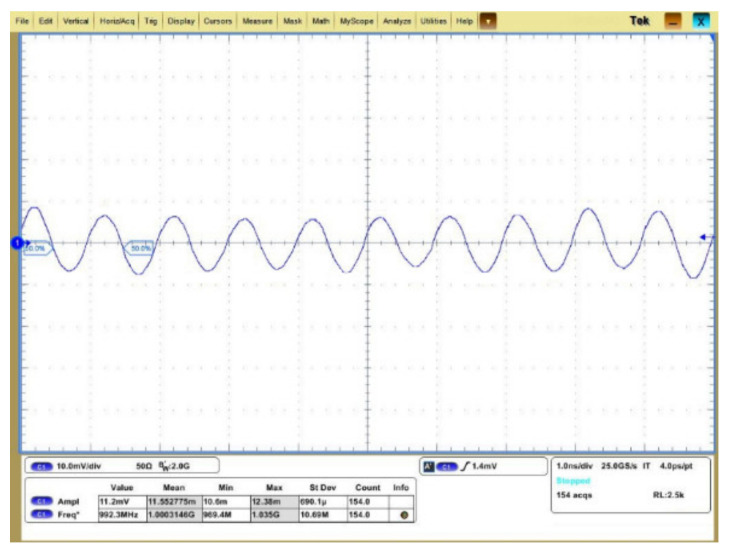
11.2-mV 1-GHz output sinusoid signal at 4.2 K.

**Figure 16:**
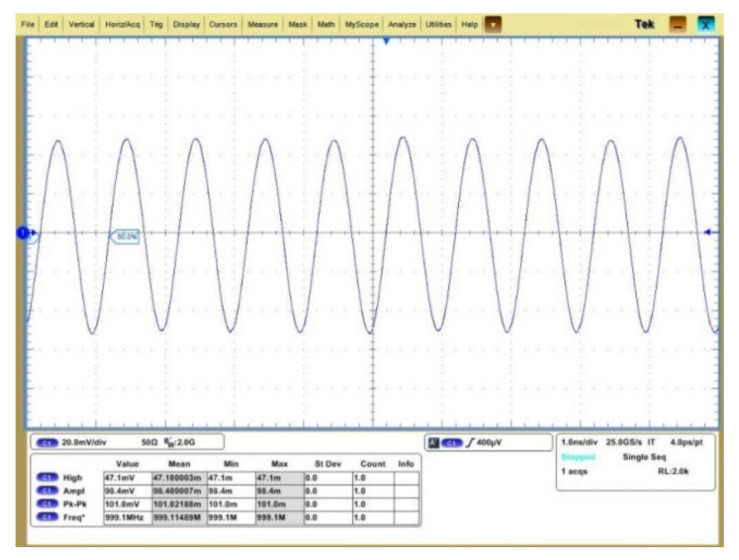
101-mV 1-GHz output sinusoid signal at 4.2 K.

As the noise performance of the cryogenic amplifier is excellent, the measurement accuracy is severely limited by the conversional noise figure (NF) measurement method [26,29]. Because of the limitation of the test equipment, it is reasonable to apply a sinusoidal stimulate signal for evaluating the amplifier noise performance, as what has been performed in [19], instead of using the cold attenuator NF test setup [26,30]. To evaluate such performance, with a 1 GHz input sinusoid signal of −60 dBm, which is the minimum signal provided by the analogue signal generator, Figure 15 shows the amplified output signal. As indicated, even when taking into account the supply noise, the noise of the measurement equipment and unwanted interferences, the output signal is clear and distinguishable at 4.2 K. With FFT calculation on the output transient signal, the calculated SNR is about 17 dB, proving that the amplifier has good SNR performance. When the input sinusoid signal increased by ten times, i.e., −50 dBm, Figure 16 shows the amplified output signal. The output signal amplitude is about 101 mV, indicating a gain compression of around 1 dB. It proves that the amplifier linearity meets the SNSPD readout requirements. 

Table 1 summarizes state-of-the-art cryogenic amplifiers operating within a several GHz frequency range [19,26,27,30,31]. For performance comparison, a figures of merit (*FOM*) defined in [19] is utilized

(8) $$FOM=\frac{\mathrm{Gain}\cdot \mathrm{BW}(\mathrm{GHz})}{P_{\mathrm{diss}}(mW)\cdot {\mathrm{Area}(\mathrm{mm}}^{2})}$$

Moreover, a new figure of merit (*FOM*_2_) adding the impact of process transition frequency *f*_T_ is defined for the performance comparison, which is calculated as:

(9) $$FOM_{2}=\frac{1}{P_{\mathrm{diss}}(mW)\cdot {\mathrm{Area}(\mathrm{mm}}^{2})}\frac{\mathrm{Gain}\cdot \mathrm{BW}(\mathrm{GHz})}{f_{T}(\mathrm{GHz})}$$

As indicated, the proposed amplifier shows competitive *FOM* and *FOM*_2_ values. Compared to the other cryogenic amplifiers, with the lowest power consumption, this work achieves good gain and bandwidth performance. To achieve optimum cryogenic amplifier performance, in [19,30] the amplifiers need to be tuned carefully. Different from these solutions, in this paper, without complex biasing tuning, the proposed amplifier achieved a robust saturated gain value, which is insensitive to temperature. Moreover, compared to [19], its power consumption is reduced by 58%.

## 5. Conclusions

To solve the lack of the accurate cryogenic device model issues and to increase robustness, by introducing a folded diode-connected transistor load, this paper proposes a temperature-insensitive wideband cryogenic amplifier for SNSPD readout applications. With a good device-tracking feature, the theoretical derivations, simulation and test results prove that the gain cell has a stable gain performance across a large temperature range. The amplifier achieves a gain of 26 dB with a bandwidth over 1 GHz at 4.2 K, consuming only 1.8 mW from a supply voltage of 1.8 V. With a 0.13 μm SiGe BiCMOS process, the chip area is only 0.5 mm^2^.

## Figures and Tables

**Table 1:** Comparison with state-of-the-art cryogenic amplifiers.

References	[19]	[26]	[27]	[30]	[31]	This Work
Technology	SiGe	SiGe	SiGe	SiGe	GaAs	SiGe
Gain (dB)	23	29.6	22	10	15	26
Bandwidth (GHz)	3.4	4.9	2.7	3.4	1.5	1
Power Dissipation (mW)	4.3	20	32	13.5	2.5	1.8
Area (mm^2^)	0.5	0.3	0.6	0.3	N/A	0.5
*FOM*	22	25	1.8	2.6	N/A	22
*f*_T_ (GHz)	210	210	200	120	N/A	210
*FOM2*	0.105	0.119	0.009	0.022	N/A	0.105

## Data Availability

Not applicable.
